# *Drosophila* Antimicrobial Peptides and Lysozymes Regulate Gut Microbiota Composition and Abundance

**DOI:** 10.1128/mBio.00824-21

**Published:** 2021-07-13

**Authors:** A. Marra, M. A. Hanson, S. Kondo, B. Erkosar, B. Lemaitre

**Affiliations:** a Global Health Institute, School of Life Science, École Polytechnique Fédérale de Lausanne (EPFL), Lausanne, Switzerland; b Invertebrate Genetics Laboratory, Genetic Strains Research Center, National Institute of Genetics, Mishima, Japan; The Scripps Research Institute; University of Hawaii at Manoa

**Keywords:** microbiota, innate immunity, aging, gnotobiotic animals, immune effectors, gut

## Abstract

The gut microbiota affects the physiology and metabolism of animals and its alteration can lead to diseases such as gut dysplasia or metabolic disorders. Several reports have shown that the immune system plays an important role in shaping both bacterial community composition and abundance in *Drosophila*, and that immune deficit, especially during aging, negatively affects microbiota richness and diversity. However, there has been little study at the effector level to demonstrate how immune pathways regulate the microbiota. A key set of *Drosophila* immune effectors are the antimicrobial peptides (AMPs), which confer defense upon systemic infection. AMPs and lysozymes, a group of digestive enzymes with antimicrobial properties, are expressed in the gut and are good candidates for microbiota regulation. Here, we take advantage of the model organism Drosophila melanogaster to investigate the role of AMPs and lysozymes in regulation of gut microbiota structure and diversity. Using flies lacking AMPs and newly generated lysozyme mutants, we colonized gnotobiotic flies with a defined set of commensal bacteria and analyzed changes in microbiota composition and abundance in vertical transmission and aging contexts through 16S rRNA gene amplicon sequencing. Our study shows that AMPs and, to a lesser extent, lysozymes are necessary to regulate the total and relative abundance of bacteria in the gut microbiota. We also decouple the direct function of AMPs from the immune deficiency (IMD) signaling pathway that regulates AMPs but also many other processes, more narrowly defining the role of these effectors in the microbial dysbiosis observed in IMD-deficient flies upon aging.

## INTRODUCTION

The gut microbiota is the complex array of microbes commonly associated with the digestive tract of animals. This bacterial consortium greatly affects host physiology, for example by promoting immune function or intestinal homeostasis (1–4). Imbalance of the microbiota, called dysbiosis, has been identified as a cause of gut dysplasia and chronic inflammatory diseases, especially during aging (5).

The fruit fly Drosophila melanogaster is a powerful model to decipher host-microbe interactions (6–8). Its genetic tractability, the possibility to generate gnotobiotic animals, and the simplicity of its natural microbiota have made Drosophila melanogaster a convenient model to gain insight into host-microbiota relationships (7, 9, 10). *Drosophila* harbors a simple gut microbiota composed of only a few dominant species, mainly belonging to *Acetobacteraceae* and *Lactobacillaceae*, which influence multiple aspects of fly physiology, such as growth (11, 12), behavior (13), life span (14), and infection resistance (15, 16). In turn, the microbiota can be shaped by various host and environmental factors, such as food composition or age of the flies (17–21).

Innate immunity is a key regulator of microbial abundance in *Drosophila* (22–25). Upon acute bacterial infection, *Drosophila* immune responsive tissues (the fat body and hemocytes in systemic infection, and epithelium in local infection) sense microbe-associated molecular patterns (MAMPs) to activate signaling pathways. In *Drosophila*, two immune pathways, the immune deficiency (IMD) and Toll pathways, regulate the expression of genes encoding immune effectors that fight invading microbes (22, 26). Studies in *Drosophila* have revealed a key role of the IMD pathway in the gut to fight pathogens and keep symbiotic bacteria in check (27–29). It is, however, unclear how the IMD pathway can effectively combat pathogens but tolerate symbiotic microbiota members in the digestive tract. In fact, the microbiota induces a low level of activation of the IMD pathway (27). Several reports have demonstrated that immune tolerance toward the indigenous microbiota is sustained by several negative feedback loops that prevent hyperactivation of the IMD pathway by peptidoglycan (the bacterial elicitor recognized by the IMD pathway) released from commensal bacteria (30–32). Compartmentalization of the immune response to restricted areas can also favor microbiota growth and control (33, 34). However, IMD pathway activation is necessary to regulate both microbiota composition and proliferation, and dysregulation of this pathway leads to abnormal bacterial growth and premature death of the host (27, 31, 32, 35). Notably, mutations affecting the IMD transcription factor Relish lead to a higher gut microbiota load and a shifted bacterial composition compared to wild-type flies (27, 36). Moreover, aged *Relish* mutant flies display dysbiosis associated with a loss of gut epithelium integrity and premature death of the animals (21, 37). Collectively, these studies point to an important role of the IMD pathway in control of the microbiota, notably during aging. However, the IMD pathway regulates hundreds of immune effectors and affects numerous physiological processes, such as enterocyte delamination and digestion (38–42). As previous studies have used mutations that suppress the whole pathway (e.g *Relish*), the precise role of individual immune effectors downstream of the IMD pathway in shaping the gut microbial community has remained elusive.

Antimicrobial peptides (AMPs) are molecules that contribute to innate defenses by targeting the negatively charged membranes of microbes (43). These peptides are produced in large quantities by the fat body during systemic infection, but also in local epithelia such as the gut. Seven classic families of inducible AMPs with several isoforms have been identified in D. melanogaster (43, 44). Use of CRISPR/Cas9 has recently enabled the generation of individual and combined AMP mutants, allowing direct investigation of their role in host defense (45). Hanson et al. showed that *Drosophila* AMPs are essential for resisting infection by Gram-negative bacteria that trigger the IMD pathway, but appear to be less involved in defense against Gram-positive bacterial infection (45).

Another key group of effector proteins that are potential regulators of Gram-positive bacteria in the gut are the lysozymes (46, 47). Lysozymes specifically cleave peptidoglycan exposed on the cell wall of Gram-positive bacteria (48). The *Drosophila* genome encodes at least 17 putative lysozymes, whose functions have never been formally addressed. Among them, six lysozyme genes (*LysB*, *D*, *E*, *P*, *S*, and *X*) are clustered in the genome at cytogenetic map position 61F. This group of lysozyme genes, notably *LysB*, *LysD*, *LysE*, and *LysP*, is strongly expressed in the digestive tract (46), and may contribute to digestive activities of the gut by degrading peptidoglycan from dietary bacteria. Furthermore, lysozyme genes are expressed in the gut upon microbiota colonization in *Drosophila*, and these proteins have been proposed to modulate immune signaling (27, 30, 49). Lysozymes may contribute to gut immunity either as direct antimicrobials, or by cleaving peptidoglycan and modulating activation of the IMD pathway (30). As such, AMPs and lysozymes may shape microbiota composition by direct interactions with microbes.

In this study, we decipher the role of two classes of antimicrobial effectors of the *Drosophila* digestive tract, the antimicrobial peptides (AMPs) and the lysozymes, on the gut microbiota. We characterized the microbiota composition in mutant flies lacking either the 14 AMP genes from seven gene families or the four gut-specific lysozyme-encoding genes in a gnotobiotic setup using 16S rRNA gene amplicon sequencing (referred to as 16S sequencing). We also assessed the role of these effectors in controlling the abundance of individual microbiota members by performing mono-association experiments. Finally, we confirmed that certain immune effectors can directly control the proliferation of microbiota members by performing systemic infections. Our findings demonstrate a direct role for both AMPs and lysozymes in controlling both the composition and abundance of the microbiota in Drosophila melanogaster.

## RESULTS

### Impact of AMPs and lysozymes on microbiota composition.

To decipher the role of AMPs and lysozymes in the regulation of gut microbiota composition, we performed 16S sequencing on gnotobiotic flies. DrosDel isogenic flies with the following genotypes were used for all experiments: the wild-type strain *w^1118^* (referred to as *w*), a compound mutant strain lacking *Defensin*, *Cecropins* (4 genes), *Drosocin*, *Diptericins* (2 genes), *Attacins* (4 genes), *Metchnikowin*, and *Drosomycin*, referred to as “Δ*AMP14*” (50), and a newly generated lysozyme-deficient mutant (referred to *LysB-P*^Δ^) (for details see the Extended Materials and Methods in Text S2 of the supplemental material). The *LysB-P*^Δ^ mutation is an 11.5-kb deletion, removing *LysC* (a putative pseudogene) and the four lysozyme genes (i.e., *Lys B*, *LysD*, *LysE*, and *Lys P*) that are known to be strongly expressed in the digestive tract (46) (Fig. S1). As expected, gut extracts from *LysB-P*^Δ^ flies have reduced lysozyme activity *ex vivo*, as monitored by their ability to digest peptidoglycan from Enterococcus faecalis (Fig. S1). We additionally included *Relish* (*Rel^E20^*) flies lacking IMD signaling as a comparative control to determine to what extent AMPs contribute to the phenotype of IMD-deficient flies.

**FIG 1:**
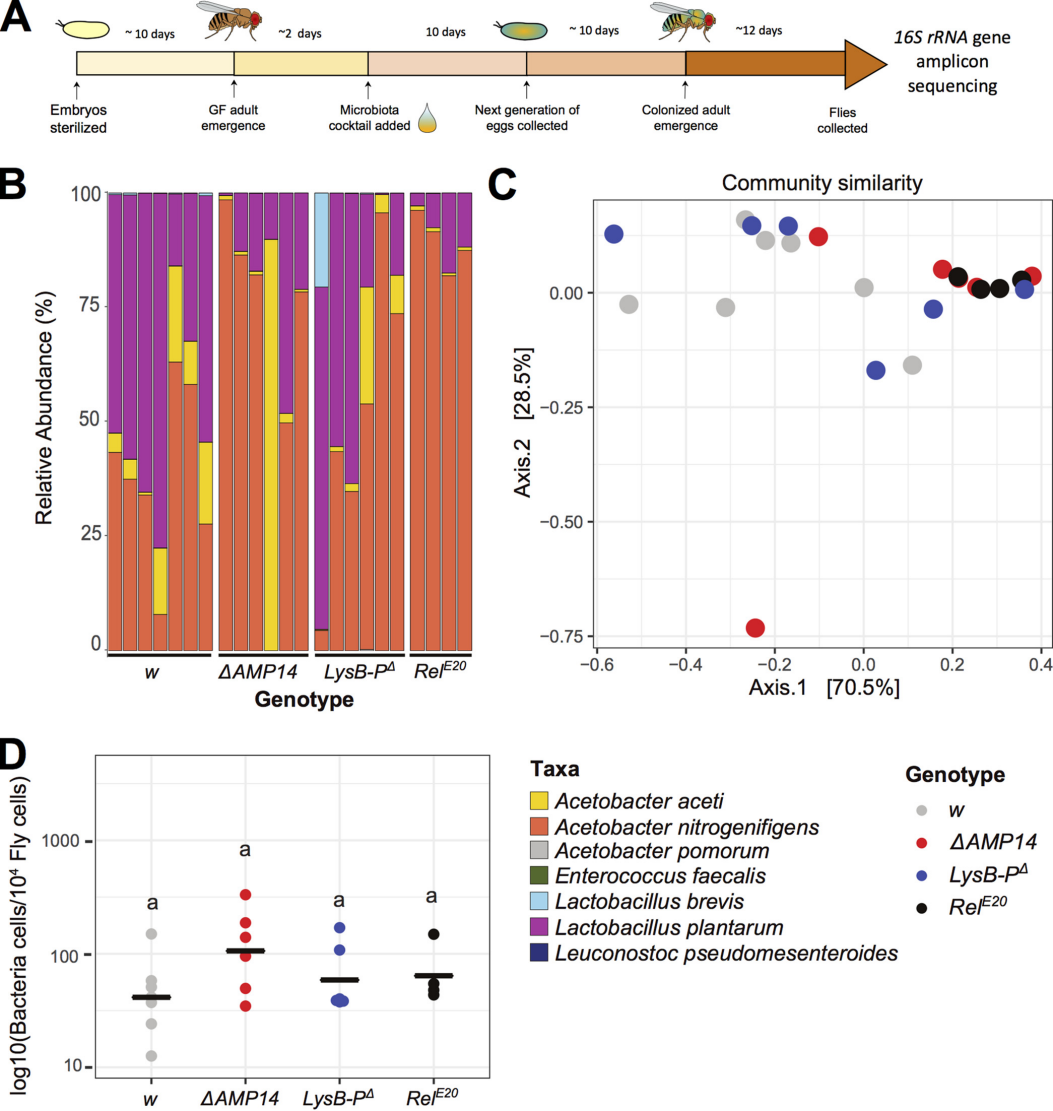
The role of AMPs and lysozymes on microbiota composition and abundance in a gnotobiotic vertical transmission setup. (A) Scheme of the experimental procedure for fly colonization and collection for 16S rRNA gene amplicon sequencing. Parental embryos were collected, sterilized in 3% bleach, and kept on antibiotic food until the adult stage. Emerging GF flies were then associated with a bacterial cocktail (microbiota cocktail) containing six representative microbiota members. Their eggs were collected over 3 days, allowed to develop to adulthood, and finally the microbiota of their adult female progeny was analyzed at ∼12 days after emergence. (B) Relative community composition of the gut microbiota in wild-type iso *w^1118^* (*w*) wild-type flies, *Relish* (*Rel^E20^*), antimicrobial peptide (Δ*AMP14*), and gut lysozyme (*LysB-P*^Δ^) mutants as determined by 16S rRNA gene amplicon sequencing. Each bar represents a biological replicate of multiple pooled flies (see Table S1 in the supplemental material for the numbers of flies included in each sample). (C) Principal coordinate analysis (PCoA) of gut communities in *w* wild-type flies, *Rel^E20^*, Δ*AMP14*, and *LysB-P*^Δ^, as determined by 16S rRNA gene amplicon sequencing. Overall colocalization of Δ*AMP14* (red dots) and *Rel^E20^* (black dots) samples and separation of these from wild-type (gray dots) samples shows that Δ*AMP14* and *Rel^E20^* samples are similar to each other and differ from wild-type samples. Stochastic distribution of *LysB-P*^Δ^ samples shows high variability in community structures between samples. (D) Absolute quantification by qPCR of the total number of bacterial cells normalized to the host gene *Actin5C.* Horizontal black bars show mean values. Details of the statistical outcomes are provided in Table S2.

**FIG 2:**
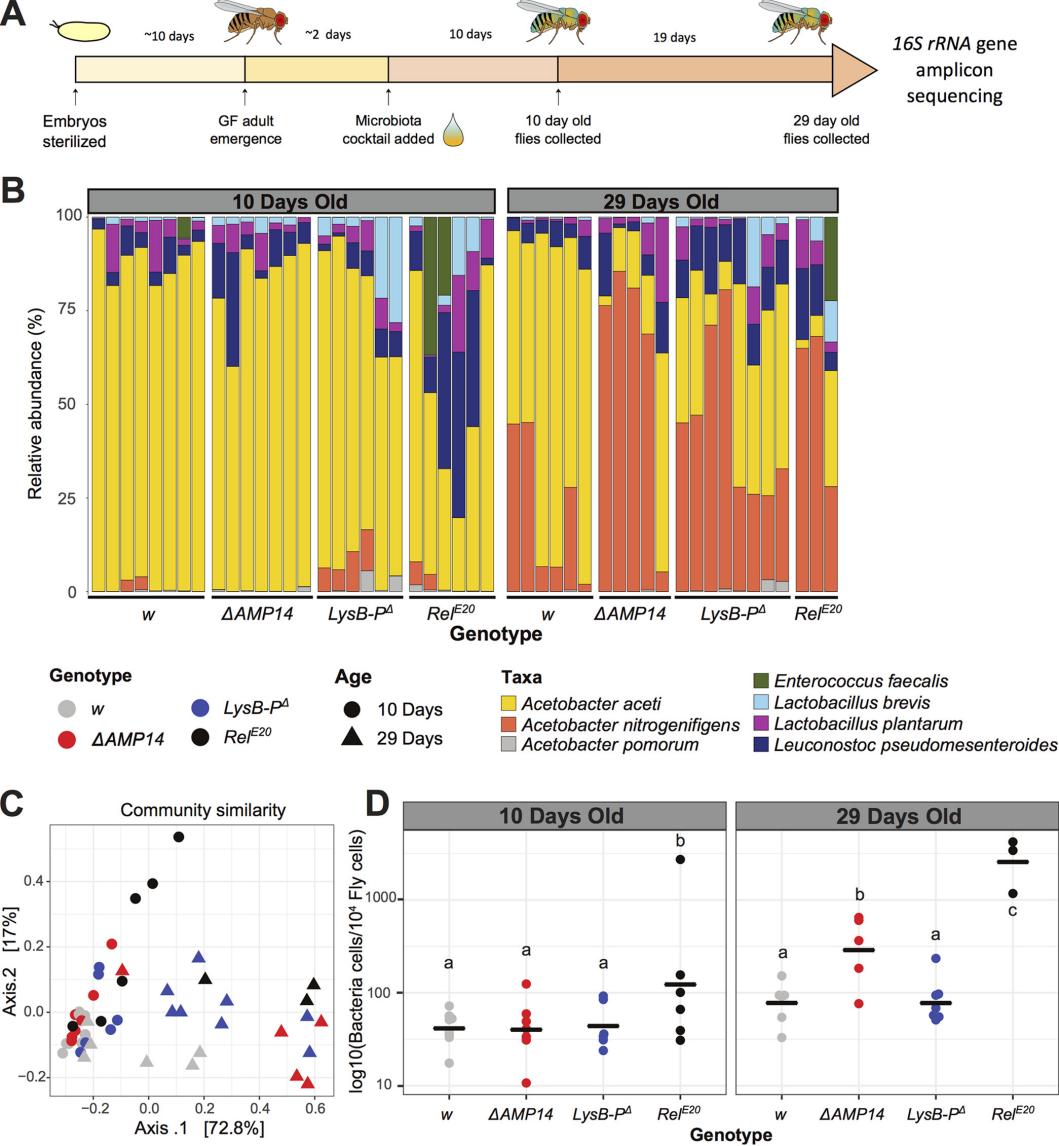
The role of AMPs and lysozymes in microbiota composition and abundance on adult microbiota in a gnotobiotic setup of young and aged flies. (A) Scheme of the experimental procedure for fly colonization and collection for 16S rRNA gene amplicon sequencing. Embryos were collected, sterilized in 3% bleach, and kept on antibiotic food until the adult stage. Emerging GF flies were associated with a bacterial cocktail containing six representative microbiota members. Females were collected for DNA extraction and 16S rRNA gene amplicon sequencing at 10 and 29 days after colonization. See Table S1 for the number of flies included in each sample. (B) Relative community composition of the gut microbiota in iso *w^1118^* (*w*) wild-type flies and *Relish* (*Rel^E20^*), antimicrobial peptide (Δ*AMP14*), and gut lysozyme (*LysB-P*^Δ^) mutants at 10 days (left panel) and 29 days (right panel) after colonization. Each bar in the plot represents a biological replicate with a pool of 5 flies each. (C) Principal coordinate analysis based on Bray-Curtis dissimilarities on the gut communities of *w* control flies, *Rel^E20^*, Δ*AMP14*, and *LysB-P*^Δ^ mutants at 10 and 29 days after colonization, based on 16S rRNA gene amplicon sequencing. Separation of the 10-day-old (dots) and 29-day-old clusters on the first axis indicates that aging is the major factor defining bacterial community composition in adults. Separation of Δ*AMP14* and *Rel^E20^* (red and black triangles) from wild-type and *LysB-P*^Δ^ (gray and blue triangles) on the same axis in the 29-day samples indicates that aging and loss of immune effectors act on microbiota composition in similar directions. (D) Absolute quantification of the total number of bacterial cells by qPCR, normalized to the host gene *Actin5C.* Horizontal black bars show mean values. Details of the statistical outcomes are provided in Table S2.

To avoid preexisting microbial community biases in different fly stocks, we performed this analysis in a gnotobiotic system with two different experimental designs. First, we analyzed the microbiota of 12-day-old flies with gut bacteria acquired through vertical transmission from gnotobiotic parental flies (i.e., germfree parents inoculated with a known community upon adult emergence) (Fig. 1A). Second, we analyzed aging-dependent changes in the adult microbiota. Here, we inoculated emerging germfree (GF) adults with a known microbiota and analyzed changes in the community structure 10 and 29 days after colonization (Fig. 2A). In this way, we uncoupled the effects of juvenile development and metamorphosis from the adult microbiota composition and abundance.

We inoculated the flies with a cocktail of six bacterial isolates that were previously described as common *Drosophila* microbiota members (19), or that were associated with the food that was used in this study (see the Materials and Methods) (8, 10, 19). These included previously characterized bacterial species as members of the *Drosophila* gut microbiota: Acetobacter pomorum (51), Lactobacillus plantarum (11), and Enterococcus faecalis (52). Our cocktail also included some incompletely characterized bacterial strains: an *Acetobacter* sp. (53), an isolate of Lactobacillus brevis, and an isolate of Leuconostoc pseudomesenteroides (see Materials and Methods, Text S1). *Acetobacter*, a genus of Gram-negative bacteria, and Lactobacillus plantarum, a Gram-positive species, both have DAP-type peptidoglycan known to activate the IMD pathway (52, 54–56). In contrast, Leuconostoc pseudomesenteroides, Lactobacillus brevis (54, 57), and Enterococcus faecalis are Gram-positive bacteria with lysine-type peptidoglycan (58), which typically activates the Toll pathway during systemic infections. Although there is no evidence for a role of the Toll pathway in the midgut (22, 42, 55), Lys-type Gram-positive bacteria can induce a basal immune reaction in the gut through the release of the metabolite uracil, which activates reactive oxygen species (ROS) production through the Duox enzyme (59).

The 16S sequencing of the six-component cocktail yielded seven amplicon sequence variants (ASVs) (60), also referred to as zero-noise operational taxonomic units (OTUs) (61) or sub-OTUS (62), across the 72 samples, with a minimum of 42,596 reads per sample after quality and abundance filtering (see the Materials and Methods and Table S1 for details). These ASVs mapped to the known species in the inoculum cocktail. Sequencing showed the *Acetobacter* sp. (53) fraction mapped to two ASVs that were distinguishable by a single nucleotide difference in their 16S amplicon. These ASVs were associated with two closely related species, Acetobacter aceti and Acetobacter nitrogenifigens (63, 64), based on their highly similar sequence.

We first focused our analysis on flies with microbiota acquired through vertical transmission from parents raised in a gnotobiotic environment (Fig. 1A, see the Materials and Methods). We found that *Rel^E20^* and *ΔAMP14* flies harbored communities dominated by *A. nitrogenifigens*, whereas the wild-type strain had a greater prevalence of *La. plantarum* (Fig. 1B). In contrast, *LysB-P^Δ^* flies had highly variable community compositions (see below), suggesting a different mode of action for these genes compared to the AMPs (Fig. 1B).

Similarities between bacterial communities were assessed using β-diversity analyses. Dissimilarities between all samples were calculated using Bray-Curtis distances plotted in a multidimensional space using principal component analysis (PCoA). This was complemented with an analysis of the dispersal (variability and spread) of the communities, and a permutation based, multivariate analysis of variance was applied to test statistical significance. These analyses showed that community compositions within *LysB-P*^Δ^ and Δ*AMP14* sample groups were more variable than the wild-type (Fig. S2A, 0.05 < *P* < 0.1), in that communities of some samples resembled wild-type flies while others resembled *Rel^E20^* flies (Fig. 1C). One *LysB-P*^Δ^ sample had a completely different profile from all other samples, with higher abundance of *La. brevis* (Fig. 1B). This suggests that the loss of AMPs or lysozymes increases stochasticity in microbiota composition. Surprisingly, communities in *Rel^E20^* mutants were more consistent between replicates, which indicates either that the stochasticity is not due to perturbation of the immune response or that the communities in these mutants stabilize earlier than in other genotypes due to other factors regulated by the IMD pathway.

In terms of community composition, distribution of data in the PCoA shows that Δ*AMP14* samples mimic *Rel^E20^* (pairwise ADONIS: *p*-adjusted_ΔAMPs vs Rel_ = 0.5), and both differ noticeably from the wild type, as demonstrated by general colocalization of Δ*AMP14* and *Rel^E20^* samples, and separation from the wild-type samples (Fig. 1C, pairwise ADONIS: *p*-adjusted_w vs Rel_ = 0.02; *p*-adjusted_w vs ΔAMPs_ = 0.06). This suggests that loss of AMPs recapitulates the effect of a general loss of the IMD pathway on the microbiota structure. As expected from the variable community composition found in *LysB-P*^Δ^ mutants (Fig. 1B), the PCoA did not reveal a distinct cluster for these samples (Fig. 1C).

Finally, we measured total bacterial loads in our samples using universal 16S rRNA gene primers (65) and *Drosophila Actin 5C* primers (53). We did not detect a statistically significant difference in total 16S rRNA gene copy numbers between the different genotypes, indicating that wild-type and mutant flies do not harbor different quantities of microbes under these conditions (Fig. 1D). Moreover, the change in microbiome variation between genotypes is not a statistical artifact of a change in absolute microbiome abundance.

Overall, our results show the microbiota composition in Δ*AMP14* flies is similar to the microbiota of *Rel^E20^* mutants that completely lack IMD signaling, suggesting that the changes in community composition observed in IMD pathway mutants is at least partly due to the specific loss of AMP production.

### Control of microbiota structure by AMPs and lysozymes during aging.

Next, we focused on the microbiota structure of adult flies that were raised in GF conditions throughout larval development and colonized only after emergence. We analyzed microbiota of these flies at both 10 and 29 days after colonization (Fig. 2A). Here, microbial communities were generally dominated by the two *Acetobacter* variants. At 10 days after colonization, *A. aceti* was the most abundant species, whereas by 29 days after colonization, *A. nitrogenifigens* was the dominant species, suggesting distinct competitive ability of the two bacteria tied to the 16S sequence variants detected in our *Acetobacter* sp. isolate.

As *Rel^E20^* mutants died earlier than other genotypes during the aging process, only three samples with fewer flies than other genotypes were included for the 29-day time point (Fig. 2B, Table S1). *Rel^E20^* mutants harbored elevated abundance of E. faecalis in 1/3 of the samples, which was not observed in other genotypes. Some samples in this genotype also had higher proportions of *La. plantarum* and *Le. pseudomesenteroides* (in two and three samples, respectively) at day 10, a trend that was not observed at day 29 (Fig. 2B). However, we cannot conclude whether this change in community structure is real or a consequence of high mortality in this genotype, leading to analysis of the survivors only. In contrast to the vertical transmission setup (Fig. S2A), *Rel^E20^* communities had high dispersion; the highest variation was observed at 10 days (Fig. S2B), and decreased at 29 days (Fig. S2C). This indicates that immunity mutations cause stochasticity in microbiota composition, but the communities are still capable of stabilizing over a long period of time.

β-Diversity and PCoA showed significant (ADONIS *P* = 0.001) separation of the 10-day-old and 29-day-old flies on the first axis, clearly pointing to aging as the major factor defining the community composition in adults (Fig. 2C). Interestingly, in 29-day-old flies, Δ*AMP14* and *Rel^E20^* were separated from wild type and *LysB-P^Δ^* mutants on the same axis (Fig. 2C), indicating that aging and loss of immune effectors act on microbiota composition in similar directions. In 10-day-old flies, we did not see similar clustering of samples except for *Rel^E20^* mutants, which were more widely dispersed on the plot (Fig. 2C). This indicates that mutations in the IMD pathway act on microbiota composition differently in young versus old flies. A statistically significant genotype × age interaction (ADONIS *P* = 0.03) supports this interpretation.

Careful examination of the relative abundance of bacteria in wild-type and mutant flies reveals interesting trends (Fig. 2B). We found that wild-type flies maintained Acetobacter aceti as the dominant *Acetobacter* ASV even after 29 days, while the proportion of lactobacilli in the community remained small. However, although Acetobacter aceti was similarly abundant at 10 days in Δ*AMP14* and *LysB-P*^Δ^ flies, Acetobacter nitrogenifigens became predominant in 29-day samples, and the proportion of lactobacilli in some samples was higher than wild type, particularly in *LysB-P*^Δ^ flies. This change in relative abundances was even more dramatic in *Rel^E20^* mutants, which were distinguished by disproportionate loads of Acetobacter nitrogenifigens and lactobacilli.

Investigation of each time point separately showed that loss of AMPs did not affect the community composition in 10-day-old flies (Fig. 2B, pairwise ADONIS, q_w vs ΔAMPs_ = 0.1). However, loss of lysozymes had detectable effects (pairwise ADONIS, q_w vs Lys_ = 0.02) on the abundance of Acetobacter pomorum, *A. nitrogenifigens*, or *La. brevis* depending on the samples, which further supports the idea of increased stochasticity in *LysB-P*^Δ^ mutants compared to wild type. This stochasticity is clearly shown by the community dispersal (Fig. S2B).

At 29 days, microbial communities in the wild type differed from those of Δ*AMP14*, *LysB-P*^Δ^, and *Rel^E20^* genotypes (pairwise ADONIS *p*-adjusted_w vs ΔAMPs_ = 0.04; *p*-adjusted_w vs Lys_ = 0.04; *p*-adjusted_w vs_ *_Rel_* = 0.04) (Fig. 2B and C). In the Δ*AMP14* strain, the relative abundance of Gram-negative *A. nitrogenifigens* consistently increased, whereas in *LysB-P*^Δ^ mutants the relative abundance of Gram-positive lactobacilli increased (Fig. 2B). This suggests that lysozymes act preferentially on Gram-positive bacteria, and the action of AMPs is limited to *Acetobacteraceae*. As all genotypes contain communities that are similarly variable (Fig. S2C), the observed differences in community composition at day 29 are unlikely to be an artifact of heterogeneity in variance among different groups.

Analysis of the total microbiota abundance showed that bacterial load differed between genotypes mainly in aged flies. At 10 days old, *Rel^E20^* flies harbored significantly larger amounts of total bacteria compared to the other genotypes, primarily due to one sample that had a high load typical in 29-day-old samples of this genotype (Fig. 2D). In 29-day-old flies, both Δ*AMP14* and particularly *Rel^E20^* flies had higher bacterial loads (Fig. 2D). These data support the notion that the IMD pathway is crucial in regulating microbiota load as the flies age and that AMPs significantly contribute to this effect of the IMD pathway.

In agreement with previous reports, our data show that microbial community composition shifts and bacterial load increases with age (17, 18, 27), and that this effect is exacerbated by loss of antimicrobials.

### Effect of AMPs and lysozymes on individual microbiota members.

**FIG 3:**
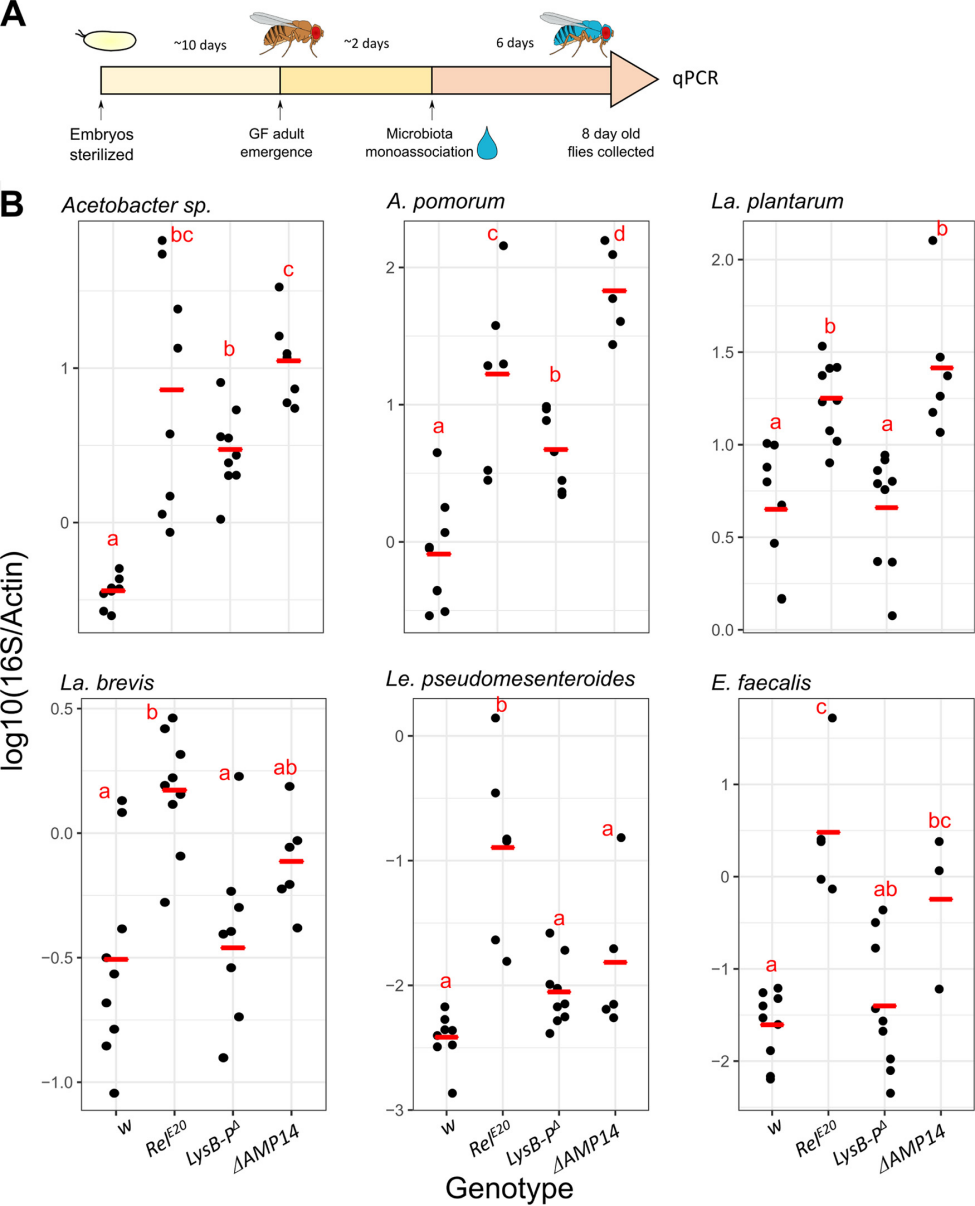
Regulation of individual microbiota members in mono-association. (A) Scheme of the experimental procedure of the mono-association experiment. Embryos were collected, sterilized in 3% bleach, and kept on antibiotic food until the adult stage. Newly emerged GF flies were then mono-associated with a single bacterial isolate. Six days after colonization, the host and bacterial DNA was extracted and qPCR analysis of the microbial load was performed. (B) Total microbial load was determined by quantitative PCR (qPCR) in female flies at 6 days after mono-association with *iso w^1118^* (*w*) wild-type flies versus *Relish* (*Rel^E20^*), antimicrobial peptide (Δ*AMP14*), and gut lysozyme (*LysB-P*^Δ^) mutant flies. Bacterial loads were assessed by qPCR with family/phylum-specific 16S rRNA gene primers and normalized to the host gene *Actin5C*. Red horizontal bars show mean values. Each dot represents a sample containing five individuals. Letters represent statistical significance (*P* < 0.05) of adjusted *P* values (FDR) from pairwise contrasts obtained from a main general linear mixed model; samples with shared letters are not statistically different from each other.

The 16S sequencing provided us a first glimpse of how AMPs and gut lysozymes regulate microbiota structure at the community level. To further characterize the effect of these antimicrobials on individual microbiota members, we used a mono-association setup where we colonized flies with each bacterial isolate from the commensal cocktail used in the 16S sequencing experiment. GF adult flies were mono-associated with a single bacterial species and the bacterial load of females was measured 6 days after colonization by quantitative qPCR (Fig. 3A). We quantified 16S rRNA gene copies using primers that recognize *Acetobacteraceae* (66) and *Firmicutes* (including *La. plantarum*, *Le. Pseudomesenteroides*, and *La. brevis*) and normalized their abundance to host cells using primers for *Actin 5C* (53) (Fig. 3B).

As expected, all mono-associated taxa established a higher load in *Rel^E20^* flies compared to wild-type flies (Fig. 3B). Interestingly, the abundance of both *Acetobacter* sp. and A. pomorum isolates was high in *LysB-P*^Δ^ but especially in Δ*AMP14* mutants (Fig. 3B), indicating that AMPs most prominently control the proliferation of these Gram-negative microbiota members. Surprisingly, in contrast to shifts toward increased lactobacilli seen in the absence of lysozymes in gnotobiotic experiments (Fig. 1, Fig. 2), mono-associated *La. plantarum* increased in abundance in the absence of AMPs but not lysozymes (Fig. 3B). This was surprising considering that lysozymes are expected to digest Gram-positive bacteria. The differing trends resulting from these approaches may depend on bacterial community dynamics in gnotobiotic experiments, or age-related differences between the experimental setups. Interestingly, Δ*AMP14* harbored significantly more E. faecalis compared to the wild-type *w* (Fig. 3B and *p*-adjusted = 0.032), and, indeed, Δ*AMP14* had bacterial abundances equivalent or even greater than *Rel^E20^* flies for all bacterial taxa except *Le. pseudomesenteroides* (Fig. 3B).

Overall, our data indicate that in the absence of bacterial community dynamics, AMPs and, to a lesser extent, lysozymes are major effectors regulating gut microbiota abundance.

### Systemic infection with microbiota members.

Previously, we showed that a lack of AMPs in the gut significantly affects the microbiota composition and growth. However, it is unclear whether AMPs have preferential antimicrobial activity that selects for core microbiota members, and to date it has not been demonstrated that AMPs directly control members of the microbiota community.

**FIG 4:**
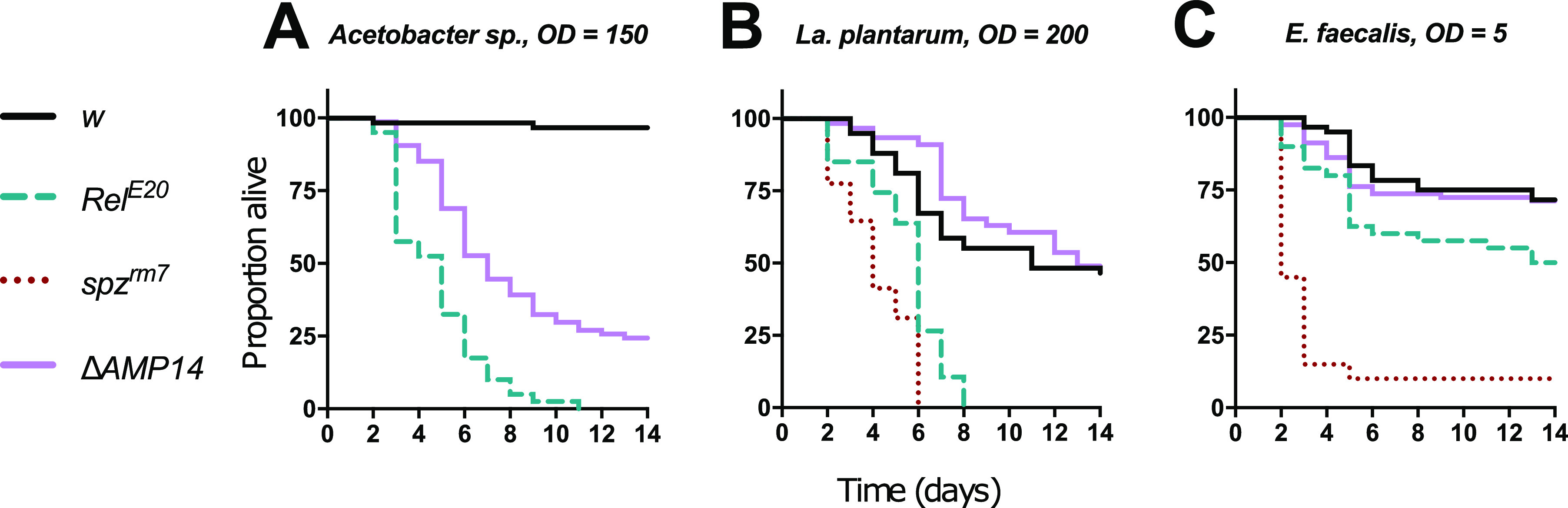
Survival upon systemic infection with microbiota bacteria. Female iso *w^1118^* wild-type flies (*w*), and *Relish* (*Rel^E20^*), antimicrobial peptide (Δ*AMP14*), and *spaetzle* (*spz^rm7^*) mutants were pricked in the thorax with three common microbiota bacteria: Gram-negative bacterium *Acetobacter* sp. (A) and two Gram-positive bacteria, *La. plantarum* (B) and E. faecalis (C). The Δ*AMP14* mutants were significantly more susceptible than wild type only to *Acetobacter* sp. infection (*P* < 0.001), and otherwise resisted infection like wild type (*P* > 0.1). Pellet densities are reported for all systemic infections as the OD_600_ value.

To address this, we used a systemic infection model to effectively “incubate” gut microbiota members in hemolymph with or without AMPs. Flies that fail to control bacterial proliferation ultimately die (67). We systemically infected flies with three representative bacteria that are normally present in the digestive tract and followed fly survival. We challenged wild-type, Δ*AMP14*, *Rel^E20^*, and *spz^rm7^* female flies by clean injury and with three different bacterial species*: Acetobacter* sp. and *La. plantarum*, which have DAP-type peptidoglycan, and E. faecalis which has Lys-type peptidoglycan. *Rel^E20^* flies lack a functional IMD response and are known to be very susceptible to systemic infection by most Gram-negative bacteria and certain classes of Gram-positive bacteria, while *spätzle^rm7^* (*spz^rm7^*) mutants lack Toll immune signaling and are susceptible to Gram-positive bacteria and fungi. We observed that Δ*AMP14* flies were more susceptible to Gram-negative *Acetobacter* sp., mimicking the susceptibility of *Rel^E20^* mutants (Fig. 4). As expected, *spz^rm7^* flies were highly susceptible to Gram-positive *La. plantarum* and E. faecalis infection, dying completely within 1 week. However, Δ*AMP14* flies did not have increased mortality when infected with these bacterial species. Flies did not die upon clean injury, indicating that the phenotype is specific to bacterial infection and is not due to a technical bias in the experiment (Fig. S3).

These systemic infections confirm that AMPs can play a direct role in the control of *Acetobacter* sp., bacteria typically found in the gut, but have a lesser impact on *La. plantarum* and E. faecalis proliferation. This trend is consistent with the results above showing that AMPs most prominently contribute to *Acetobacter* control after gnotobiotic or mono-associative colonization.

## DISCUSSION

In *Drosophila*, the immune system, and particularly the IMD pathway, has been robustly demonstrated to be an important regulator of the gut microbiota and intestinal homeostasis (21, 22, 27, 29, 32, 36). Several reports have indicated the importance of the IMD pathway in maintaining balanced microbiota during aging, and mutants for this pathway (e.g *Relish*) have atypical microbiota abundance and composition (27). While it is clear the IMD pathway is a regulator of the gut microbiota, little is known about the effectors mediating this regulation. In addition to regulating most AMP expression in the gut (21), the IMD pathway regulates other physiological aspects, including expression of digestive enzymes (41) and enterocyte delamination (39, 68, 69). The present work extends these studies by more narrowly defining the role of AMPs and lysozymes by comparing specific loss of these effectors to total loss of IMD signaling.

Our results confirm a prominent role for the IMD pathway in regulating both microbiota load and diversity, especially upon aging (21, 27, 36). The Δ*AMP14* genotype mimics the *Relish* phenotype in many respects, showing that AMPs indeed contribute downstream of IMD to shape the microbiota composition. Our experiments consistently showed an increase in the load of *Acetobacter* species in both *Rel^E20^* and Δ*AMP14* flies. The observation that both Δ*AMP14* and *Rel^E20^* flies are also susceptible to *Acetobacter* systemic infection, together with previous studies showing that AMPs contribute to survival upon Gram-negative bacterial infection (45, 50), provides strong evidence that direct microbicidal activity of AMPs regulates these Gram-negative bacteria. Collectively, this indicates for the first time that the basal level of IMD pathway activity induced by the gut microbiota (27) leads to the production of AMPs that prevent overgrowth of Gram-negative commensals, such as *Acetobacter*. Future studies should clarify which AMP(s) among the 14 deleted in the Δ*AMP14* flies regulate *Acetobacter.*

*La. plantarum* is an important member of *Drosophila* microbiota that is associated with the host, both in larval development and adulthood (11, 33, 70). As the DAP-type peptidoglycan found in the cell wall of these bacteria can activate the IMD pathway, we might expect to see an action of AMPs against them. However, the IMD pathway and AMP mutants are not very susceptible to DAP-type Gram-positive bacteria (45, 50). Moreover, d-alanylation of *La. plantarum* lipoteichoic acid has recently been proposed as a mechanism to protect against the action of AMPs and lysozymes (49). Here, we found that the AMPs play a role in controlling *La. plantarum* abundance in a mono-colonization setup but not when bacteria are in a community context. This might be due to the dynamics between microbiota members (e.g., competition between different species) or due to differential affinity of AMPs for the peptidoglycan of distinct species. It is possible that the abundance of *La. plantarum* is maintained at a threshold level in the gut and this is naturally achieved in a community through bacterial interactions. However, *La. plantarum* overgrowth can be inhibited by AMPs in a context where it becomes the only dominant member of the community.

Other factors that could influence the microbiota include developmental effects and sex-specific effects on immunity and nutrition (71, 72). Here, we only assessed the microbiota of adult females, but future studies should confirm if these AMP-microbiota interactions are consistent in other conditions. It should also be noted that we transferred the microbiota directly using beads, which could affect microbial community structure, e.g., by accruing microbial load over time or by avoiding bottleneck events that could have exacerbated stochastic microbe species takeover or drop out. We also used both propionic acid and methylparaben sodium salt (Moldex) in our food medium. These two preservative compounds are routinely used to prevent fly food spoilage, though it is known that they can affect microbial growth (73). Future investigations would benefit from monitoring microbial loads in the food while manipulating microbiota transfer techniques and fly diet.

The genome of *Drosophila* contains many genes encoding lysozymes, likely as a consequence of living in bacterially enriched habitats (46). Indeed, animals feeding on fermenting medium, such as ruminants or fruit flies, have a much higher number of lysozyme gene copies compared to animals feeding on “clean food” (74, 75). In many insects, lysozymes are induced upon systemic infection, pointing to a possible role as immune effectors. In contrast, *Drosophila* lysozymes are strongly expressed in the gut, indicating a specific role in the digestive process (46, 47). Of note, one uncharacterized gene annotated as encoding a putative lysozyme (*CG6429*) is strongly induced upon systemic infection and is partially regulated by the IMD pathway (38).

In this study, we generated a *LysB-P*^Δ^ mutant deficient for four lysozyme genes strongly expressed in the gut. *LysB-P*^Δ^ gut extracts have reduced lysozyme activity (Fig. S1), confirming that these four genes indeed contribute to gut lysozyme activity. As lysozymes are known to digest peptidoglycan and can exhibit bactericidal activity alone or in combination with AMPs (48), we were interested to monitor the impact of lysozymes on the gut microbiota. We expected that loss of lysozymes would have a greater effect on Gram-positive bacteria, as the thin peptidoglycan layer of Gram-negative bacteria is protected by their external lipopolysaccharide (LPS) membrane. Consistently, 16S sequencing revealed that *LysB-P*^Δ^ mutants exhibited increased relative community *Lactobacillus* abundance. However, mono-association experiments revealed a role of lysozymes in suppressing growth of only Gram-negative *Acetobacter* species. This effect was less marked than that of AMP deficient mutants.

An interesting observation of our study is that flies lacking AMPs or lysozymes displayed greater community stochasticity, similar to the phenotype of *Rel^E20^* flies. This suggests that multiple factors, including AMPs, lysozymes, and bacteria-bacteria interactions, contribute to stability of the gut microbiota, and that loss of these factors increases stochasticity. We avoided complications of fly genetic backgrounds by using isogenic fly strains. While the isogenization process homogenizes the genetic background, it also increases the degree of homozygosity along the genome, with a possible increase in genetic interactions. Thus, our study on AMPs and lysozymes using the *iso Drosdel* background should be reinforced by other studies using other backgrounds or alternative approaches.

In *Drosophila*, the induction of genes for antibacterial peptides after infection is blocked in IMD pathway mutants, such as *Rel^E20^*, resulting in high susceptibility. These flies also cannot control their microbiota load, especially during aging (27). As expected, we found similar gut microbiota structure in *Relish* and *AMP* mutants. Indeed, both genotypes were unable to control the microbiota load and composition, but *Rel^E20^* flies had a more severe phenotype, with 16S analysis showing atypical microbial composition at early life stages and marked inability to control all inoculated bacterial species in mono-association experiments. This is likely due to the multiple roles of the IMD pathway in gut physiology, apoptosis, nutrition, and metabolism (39, 76, 77), the loss of which, in addition to AMPs, may exacerbate gut dysbiosis or hasten the inability of the flies to control microbiota growth. This indicates that although AMPs play an important role in control of microbiota members, they contribute only partially to the dysbiosis of mutant flies with perturbed IMD pathways.

Collectively, our work is the first to show direct involvement of AMPs and lysozymes in the control of *Drosophila* gut microbiota. Consequences of the loss of these effectors are exacerbated during aging, and their loss contributes to increased microbiota abundance and shifted composition. This work shows that immune effectors typically associated with resistance to pathogenic infections also help shape the beneficial gut community, consistent with the idea that host-symbiont interactions use the same “language” typically associated with pathogenesis (78).

## MATERIALS AND METHODS

### Bacterial strains and culture conditions.

Bacterial strains used in this study and their origins are as follows: *Acetobacter* sp. (53), Acetobacter pomorum (51), Lactobacillus plantarum (11), and Enterococcus faecalis (52). Lactobacillus brevis and Leuconostoc pseudomesenteroides were isolated from the “Valais” population, collected in the Valais canton of Switzerland in 2007 (79). Briefly, homogenates from 20 flies were spread over Man, Rogosa and Sharpe (MRS)-d-Mannitol 2.5% plates. A single colony was used to prepare liquid cultures (described below) and establish glycerol stocks, as well as for 16S rRNA gene full-length amplification using universal primers. The PCR products were sequenced by Sanger sequencing and assigned to taxa based on a Microbial BLASTn search against the nucleotide database of NCBI (https://blast.ncbi.nlm.nih.gov/Blast.cgi?PAGE_TYPE=BlastSearch&BLAST_SPEC=MicrobialGenomes). See Text S1 in the supplemental material for full 16S rRNA gene sequences of these isolates and *Acetobacter* sp. (53).

A. pomorum and *Acetobacter* sp. were cultivated in (MRS)-d-Mannitol 2.5% medium in aerobic conditions at 29°C for at least 18 h with agitation. *La. plantarum*, *La. brevis*, and *Le. pseudomesenteroides* were cultivated in anaerobic conditions in (MRS)-d-Mannitol 2.5% medium at 29°C for at least 18 h standing. E. faecalis was cultured in brain heart infusion (BHI) broth medium in aerobic conditions at 37°C for at least 16 h with agitation.

### Gnotobiotic fly cultivation and media.

All experiments were done at 25°C with a 12-h dark/light cycle. Embryos were collected and emerging adults were a mix of males and females. Only the microbiotas of females were assessed. Because antibiotic treatments over multiple generations may result in epigenetic effects that may interfere with phenotypes (80–84), GF flies were freshly generated for each experiment. Please see the Extended Methods for details.

To avoid sticky “biofilm-like” formation on the medium, flies were transferred to fresh medium every 3 to 4 days. To avoid a decrease in microbial loads, the microbiota of each tube was transferred to the next using glass beads (Fig. S4). Briefly, flies were anaesthetized on ice and removed on sterile caps. Then, 10 to 20 glass beads were transferred to the old tube and shaken for 10 s. The beads were then transferred to the new sterile tube and were shaken again for 10 s to spread the bacteria around the tubes. Adults were then added in the new tubes. For 16S RNA-seq experiments, flies were sampled at 10 days and 29 days after colonization taken at least 3 days after the last transfer to fresh food.

For vertically transmitted microbiota, we let gnotobiotic adults (colonized as described above 3 to 4 days previously) lay eggs on fresh medium for 3 days. We let larvae grow in this original medium and began transferring the emerging adults to new tubes 5 days after the first fly emerged. We collected adults 10 to 15 days after emergence and analyzed their associated bacterial community.

For mono-association experiments, we colonized flies with each isolate that was included in the commensal cocktail. Because during mono-association changes in community structure are not a concern, we maintained the flies in their original tube throughout the experiment. Flies were sampled 6 days after colonization.

### DNA extraction and qPCR.

Surface-sterilized flies (washed in sterile water and EtOH) were mechanically lysed by bead beating and the DNA extraction was carried out on samples using a DNeasy blood and tissue kit (Qiagen).

The qPCRs for absolute quantification were carried out as previously described (85) and for relative quantification qPCRs as described in reference 66. The universal (85) and *Acetobacter*-specific 16S (66) and Actin 5C primers (66) were previously described. *Firmicutes*-specific primers (antisense primer 5′-AGCGTTGTCCGGATTTAT-3′, sense primer 5′-CATTTCACCGCTACACAT-3′) were designed by aligning the 16S rRNA gene sequences of the four *Firmicute*s species that were used in this study. Their specificity (lack of amplification in *Acetobacteraceae*) was determined on plasmid DNA containing specific 16S sequences, as well as on DNA extracted from flies mono-associated with microbiota members described in this study.

### 16S rRNA gene amplicon sequencing and data processing.

Amplification of the V4 region of the 16S rRNA gene and the library preparation protocol were done as previously described (85), except that the first cycle of PCR was performed with 30 cycles. Libraries were verified by Fragment analyzer, mixed with 10% PhiX library (Illumina number FC-110-3001), and subjected to Illumina MiSeq v3 paired-end sequencing in one lane, with all libraries multiplexed.

Sequencing data have been processed using Divisive Amplicon Denoising Algorithm 2 (DADA2) pipeline (“dada2” package version 1.14.1 in R) and “phyloseq” package version 1.30.0. Please see the Extended Methods for further details.

### Diversity and statistical analysis.

Permutational multivariate analysis of variance (ADONIS, “adonis” function) based on Bray–Curtis distances (“vegdist” function) (86) was used to test the effects of age and genotype on community structure, and “metaMDS” function was used for plotting beta-diversity. For pairwise comparisons of ADONIS, “adonis.pair” function was used from the “EcolUtils” package. To test the dispersion of communities, we used the function “betadisper” (87, 88) and compared the distances of individual samples to group centroids in multidimensional space using “permutest.”

All statistical analyses were performed using R (version 3.6.3). We used general linear mixed models (“lme4” package version 1.1.23) to test for the effects of age, genotype, and their interaction (depending on the experimental design) on bacterial loads or dispersion of bacterial communities. Pairwise comparisons were performed using “emmeans” and “pairs” functions (“emmeans” package version 1.5.1). *P* values were adjusted using the FDR method.

### Systemic infection and life span assay.

Systemic infections were performed by pricking 5- to 7-day-old conventionally reared adult females in the thorax with a 100-μm-thick insect pin dipped into a concentrated pellet of bacteria. The bacteria were grown to the following concentration values measured by the optical density at 600 nm (OD_600_) in (MRS)-d-Mannitol 2.5% broth (*Acetobacter* sp., OD_600_ = 150; *La. plantarum*, OD_600_ = 200) or BHI (E. faecalis, OD_600_ = 5). Infected flies were maintained at 25°C for experiments. At least three replicate survival experiments were performed for each infection, with 20 flies per vial on standard fly medium without yeast. Survivals were scored daily and flies were moved to fresh medium every 2 days.

Survival data were analyzed using the survival package in R 3.6.3 with a Cox proportional hazards model (coxph() function) including experiment as a covariate in the final model, when significant.

### Data availability.

16S rRNA gene amplicon sequencing data have been submitted to the NCBI database under BioProject no. PRJNA742915.

10.1128/mBio.00824-21.1 FIG S1 Lysozyme mutant characterization. (A) Diagram of the *LysB-P*^Δ^ mutation, which deletes 11.5 kb of nucleotide sequence from the *Drosophila* genome. *LysB-P*^Δ^ flies lack the four lysozyme genes (*LyB, LysD, LysE,* and *LysP*) that are highly expressed in the gut, along with the *LysC* pseudogene. (B) *LysB-P*^Δ^ mutants have a reduced capacity to degrade peptidoglycan in the gut compared to wild-type flies. Gut extracts from wild-type flies (*w*) and lysozyme mutants (*LysB-P*^Δ^) were incubated with E. faecalis peptidoglycan for 48 h. The lysozyme activity was monitored by change in optical density of the peptidoglycan solution. Commercial lysozyme was included as a positive control (See the Extended Methods for details). Download FIG S1, EPS file, 0.1 MB. Copyright © 2021 Marra et al. 2021 Marra et al. https://creativecommons.org/licenses/by/4.0/ This content is distributed under the terms of the Creative Commons Attribution 4.0 International license.

10.1128/mBio.00824-21.2 FIG S2 Homogeneity of multivariate dispersals in microbial communities. Distance to the center of each group (genotype) represents the spread of the communities in the multivariate space (PCoA) in a vertical transmission setup (A), 10 days after colonization (B), and 29 days after colonization (C). Different letters indicate significant differences (*P* < 0.05) between *P* values upon pairwise comparison, calculated by permutations on multivariate equivalent of variances. A significant difference between groups indicates that samples belonging to one group are more variable in microbiota composition compared to the other group. Download FIG S2, EPS file, 0.2 MB. Copyright © 2021 Marra et al. 2021 Marra et al. https://creativecommons.org/licenses/by/4.0/ This content is distributed under the terms of the Creative Commons Attribution 4.0 International license.

10.1128/mBio.00824-21.3 FIG S3 Δ*AMP14* flies survive clean injury. Females from the indicated genotypes were pricked with a clean 100-μm insect pin. No genotype displayed pronounced susceptibility to clean injury. Download FIG S3, EPS file, 0.1 MB. Copyright © 2021 Marra et al. 2021 Marra et al. https://creativecommons.org/licenses/by/4.0/ This content is distributed under the terms of the Creative Commons Attribution 4.0 International license.

10.1128/mBio.00824-21.4 FIG S4 Microbiota transfer with beads. See detailed protocol in the Materials and Methods in the section “Gnotobiotic fly cultivation and media.” FIG S4, TIF file, 0.4 MB Copyright © 2021 Marra et al. 2021 Marra et al. https://creativecommons.org/licenses/by/4.0/ This content is distributed under the terms of the Creative Commons Attribution 4.0 International license.

10.1128/mBio.00824-21.5 TEXT S1 16S rRNA gene sequences of *La. brevis*, *Le. pseudomesenteroides*, and *Acetobacter sp*. (see Erkosar et al., 2017) used in this study. Download Text S1, DOCX file, 0.01 MB. Copyright © 2021 Marra et al. 2021 Marra et al. https://creativecommons.org/licenses/by/4.0/ This content is distributed under the terms of the Creative Commons Attribution 4.0 International license.

10.1128/mBio.00824-21.6 TABLE S1 Number of flies used in each sample and number of reads that were retained after each step of the 16S rRNA gene analysis. Download Table S1, XLSX file, 0.02 MB. Copyright © 2021 Marra et al. 2021 Marra et al. https://creativecommons.org/licenses/by/4.0/ This content is distributed under the terms of the Creative Commons Attribution 4.0 International license.

10.1128/mBio.00824-21.7 TABLE S2 Summary of the statistics used for the data analyzed in Fig. 1 and Fig. 2. Download Table S2, XLSX file, 0.02 MB. Copyright © 2021 Marra et al. 2021 Marra et al. https://creativecommons.org/licenses/by/4.0/ This content is distributed under the terms of the Creative Commons Attribution 4.0 International license.

10.1128/mBio.00824-21.8 TEXT S2 Extended Materials and Methods. Supplementary information about *Drosophila* genetics, 16S data analysis, gnotobiotic fly cultivation, and lysozyme peptidoglycan digestion assay. Download Text S2, DOCX file, 0.03 MB. Copyright © 2021 Marra et al. 2021 Marra et al. https://creativecommons.org/licenses/by/4.0/ This content is distributed under the terms of the Creative Commons Attribution 4.0 International license.
