# Supplementary figures and images for: Drosophila Antimicrobial Peptides and Lysozymes Regulate Gut Microbiota Composition and Abundance
Source: mBio. 2021 Jul 13;12(4):e00824-21. doi: 10.1128/mBio.00824-21 (PMC8406169; doi:10.1128/mBio.00824-21)

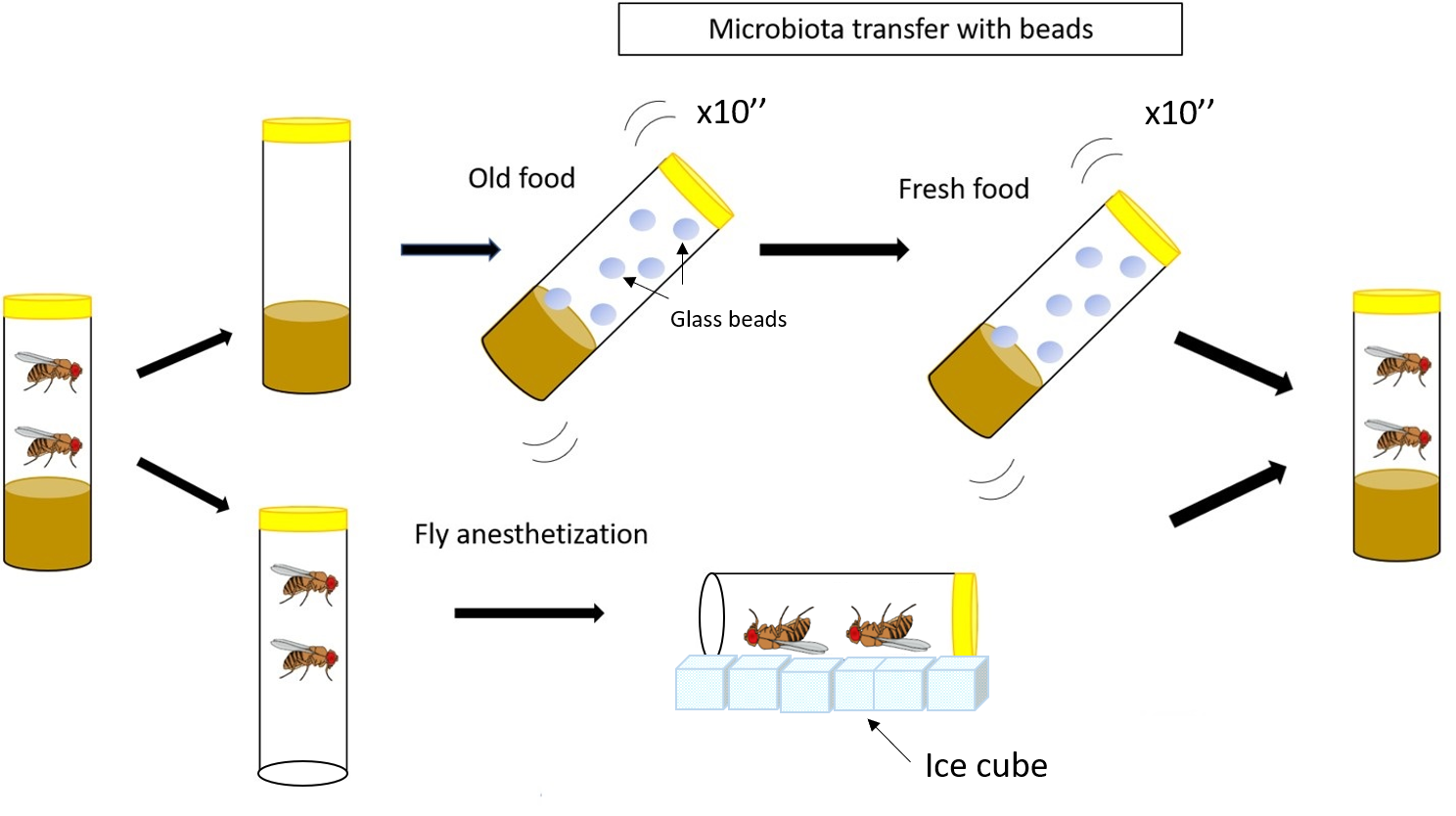

Supplement: FIG S4 [file mbio.00824-21-sf004.tif]
