# Supplementary material for: Drosophila Antimicrobial Peptides and Lysozymes Regulate Gut Microbiota Composition and Abundance
Source: mBio. 2021 Jul 13;12(4):e00824-21. doi: 10.1128/mBio.00824-21 (PMC8406169; doi:10.1128/mBio.00824-21)
Supplement: TEXT S1 [file mbio.00824-21-t0001.docx]

**Supplementary Text 1:** 16S rRNA gene sequences of La. brevis, *Le. pseudomesenteroides* and *Acetobacter sp*. [REF erkosar 2017] used in this study.

>Lactobacillus_brevis_16S

CCTAATACATGCAAGTCGAACGAGCTTCCGTTGAATGACGTGCTTGCACTGATTTCAACAATGAAGCGAGTGGCGAACTGGTGAGTAACACGTGGGGAATCTGCCCAGAAGCAGGGGATAACACTTGGAAACAGGTGCTAATACCGTATAACAACAAAATCCGCATGGATTTTGTTTGAAAGGTGGCTTCGGCTATCACTTCTGGATGATCCCGCGGCGTATTAGTTAGTTGGTGAGGTAAAGGCCCACCAAGACGATGATACGTAGCCGACCTGAGAGGGTAATCGGCCACATTGGGACTGAGACACGGCCCAAACTCCTACGGGAGGCAGCAGTAGGGAATCTTCCACAATGGACGAAAGTCTGATGGAGCAATGCCGCGTGAGTGAAGAAGGGTTTCGGCTCGTAAAACTCTGTTGTTAAAGAAGAACACCTTTGAGAGTAACTGTTCAAGGGTTGACGGTATTTAACCAGAAAGCCACGGCTAACTACGTGCCAGCAGCCGCGGTAATACGTAGGTGGCAAGCGTTGTCCGGATTTATTGGGCGTAAAGCGAGCGCAGGCGGTTTTTTAAGTCTGATGTGAAAGCCTTCGGCTTAACCGGAGAAGTGCATCGGAAACTGGGAGACTTGAGTGCAGAAGAGGACAGTGGAACTCCATGTGTAGCGGTGGAATGCGTAGATATATGGAAGAACACCAGTGGCGAAGGCGGCTGTCTAGTCTGTAACTGACGCTGAGGCTCGAAAGCATGGGTAGCGAACAGGATTAGATACCCTGGTAGTCCATGCCGTAAACGATGAGTGCTAAGTGTTGGAGGGTTTCCGCCCTTCAGTGCTGCAGCTAACGCATTAAGCACTCCGCCTGGGGAGTACGACCGCAAGGTTGAAACTCAAAGGAATTGACGGGGGCCCGCACAAGCGGTGGAGCATGTGGTTTAATTCGAAGCTACGCGAAGAACCTTACCAGGTCTTGACATCTTCTGCCAATCTTAGAGATAAGACGTTCCCTTCGGGGACAGAATGACAGGTGGTGCATGGTTGTCGTCAGCTCGTGTCGTGAGATGTTGGGTTAAGTCCCGCAACGAGCGCAACCCTTATTATCAGTTGCCAGCATTCAGTTGGGCACTCTGGTGAGACTGCCGGTGACAAACCGGAGGAAGGTGGGGATGACGTCAAATCATCATGCCCCTTATGACCTGGGCTACACACGTGCTACAATGGACGGTACAACGAGTTGCGAAGTCGTGAGGCTAAGCTAATCTCTTAAAGCCGTTCTCAGTTCGGATTGTAGGCTGCAACTCGCCTACATGAAGTTGGAATCGCTAGTAATCGCGGATCAGCATGCCGCGGTGAATACGTTCCCGGGCCTTGTACACACCGCCCGTCACACCATGAGAGTTTGTAACACCCAAAGCCGGTGAGATAACCTTCGGGAGTCAGCCGTCTAAGGGACGAT

>Leuconostoc_pseudomesenteroides_16S

GCTAATACATGCAAGTCGAACGCACAGCGAAAGGTGCTTGCACCTTTCAGTGAGTGGCGAACGGGTGAGTAACACGTGGACAACCTGCCTCAAGGCTGGGGATAACATTTGGAAACAGATGCTAATACCGAATAAAACTCAGTGTCGCATGACACAAAGTTAAAAGGCGCTTTGGCGTCACCTAGAGATGGATCCGCGGTGCATTAGTTAGTTGGTGGGGTAAAGGCCTACCAAGACAATGATGCATAGCCGAGTTGAGAGACTGATCGGCCACATTGGGACTGAGACACGGCCCAAACTCCTACGGGAGGCTGCAGTAGGGAATCTTCCACAATGGGCGAAAGCCTGATGGAGCAACGCCGCGTGTGTGATGAAGGCTTTCGGGTCGTAAAGCACTGTTGTATGGGAAGAACAGCTAGAATAGGGAATGATTTTAGTTTGACGGTACCATACCAGAAAGGGACGGCTAAATACGTGCCAGCAGCCGCGGTAATACGTATGTCCCGAGCGTTATCCGGATTTATTGGGCGTAAAGCGAGCGCAGACGGTTGATTAAGTCTGATGTGAAAGCCCGGAGCTCAACTCCGGAATGGCATTGGAAACTGGTTAACTTGAGTGCAGTAGAGGTAAGTGGAACTCCATGTGTAGCGGTGGAATGCGTAGATATATGGAAGAACACCAGTGGCGAAGGCGGCTTACTGGACTGTAACTGACGTTGAGGCTCGAAAGTGTGGGTAGCAAACAGGATTAGATACCCTGGTAGTCCACACCGTAAACGATGAACACTAGGTGGTTAGGAGGTTTCCGCCTCTTAGTGCCGAAGCTAACGCATTAAGTGTTCCGCCTGGGGAGTACGACCGCAAGGTTGAAACTCAAAGGAATTGACGGGGACCCGCACAAGCGGTGGAGCATGTGGTTTAATTCGAAGCAACGCGAAGAACCTTACCAGGTCTTGACATCCTTTGAAGCTTTTAGAGATAGAAGTGTTCTCTTCGGAGACAAAGTGACAGGTGGTGCATGGTCGTCGTCAGCTCGTGTCGTGAGATGTTGGGTTAAGTCCCGCAACGAGCGCAACCCTTATTGTTAGTTGCCAGCATTCAGATGGGCACTCTAGCGAGACTGCCGGTGACAAACCGGAGGAAGGCGGGGACGACGTCAGATCATCATGCCCCTTATGACCTGGGCTACACACGTGCTACAATGGCGTATACAACGAGTTGCCAACCCGCGAGGGTGAGCTAATCTCTTAAAGTACGTCTCAGTTCGGATTGTAGTCTGCAACTCGACTACATGAAGTCGGAATCGCTAGTAATCGCGGATCAGCACGCCGCGGTGAATACGTTCCCGGGTCTTGTACACACCGCCCGTCACACCATGGGAGTTTGTAATGCCCAAAGCCGGTGGCCTAACCTTTAGGAAGGAGCCGTCTAAGGCAGACCG

>Acetobacter_sp_16S

atGCaGTcgcanGaaGgCTTCGGCCTTAGTGGCGGacGGGTGAGTAACGCGTAGGAATCTATCCatGGgtGGGGGATAACTCCGGGAAACTGGAGctAATACCGCATGATACCTGAGGGTCAAAGGCGCAAGTCGCCTgtGGAGGAGCCTGCGTTTGATTAGCTTGTTGGTGGGGTAAAGGCCTACCAAGGCGATGATCAATAGCTGGTCTGAGAGGATGATCAGCCACACTGGGACTGAGACACGGCCCAGACTCCTACGGGAGGCAGCAGTGGGGAATATTGGACAATGGGGGAAACCCTGATCCAGCAATGCCGCGTGTGTGAAGAAGGTTTTCGGATTGTAAAGCACTTTCGGCGGGGACGATGATGACGGTACCCGCAGAAGAAGCCCCGGCTAACTTCGTGCCAGCAGCCGCGGTAATACGAAGGGGGCTAGCGTTGCTCGGAATGACTGGGCGTAAAGGGCGTGTAGGCGGTTTGTACAGTCAGATGTGAAATCCCCGGGCTTAACCTGGGAGCTGCATTTGATACGTGCAGACTAGAGTATGAGAGAGGGTTGTGGAATTCTCAGTGTAGAGGTGAAATTCGTAGATATTGGGAAGAACACCGGTGGCGAAGGCGGCAACCTGGCTCATTACTGACGCTGAGGCGCGAAAGCGTGGGGAGCAAACAGGATTAGATACCCTGGTAGTCCACGCTGTAAACGATGTGTGCTGGATGTTGGGTAACTTAGTTACTCAGTGTCGTAGCTAACGCGATAAGCACACCGCCTGGGGAGTACGGCCGCAAGGTTGAACTCAAAGGAATTGACGGGGGCCCGCACAAGCGGTGGAGCATGTGGTTTAATTCGAAGCAACGCGCAGAACCTTACCAGGGCTTGTATGGAGAGGCTGTATTCAGAGATGGATATTTCCCGCAAGGGACCTCTTGCACAGGTGCTGCATGGCTGTCGTCAGCTCGTGTCGTGAGATGTTGGGTTAAGTCCCGCAACGAGCGCAACCCTTATCTTTAGTTGCCAGCATGTTTGGGTGGGCACTCTAAAGAGACTGCCGGTGACAAGCCGGAGGAAGGTGGGGATGACGTCAAGTCCTCATGGCCCTTATGTCCTGGGCTACACACGTGCTACAATGGCGGTGAcagTGGGAAGCTAGATGGTGACATCATGCCGATCTCTAAAAACCGTCTCAGTTCGGATTGCACTCTGCAACTCGAGTGCATGAAGGTGGAATCGCTAGTAATCGCGGATCAGcatGCCGCGGTGAATACGTTCCCGGGCCTTGTACAcacCGCCCGTCACaccATGGGAGTTGGnTnaCCTTAAGCCgGTgaGCGAACCgcaaGAcgcaAGcgGa
