# Supplementary material for: Drosophila Antimicrobial Peptides and Lysozymes Regulate Gut Microbiota Composition and Abundance
Source: mBio. 2021 Jul 13;12(4):e00824-21. doi: 10.1128/mBio.00824-21 (PMC8406169; doi:10.1128/mBio.00824-21)
Supplement: TEXT S2 [file mbio.00824-21-t0002.docx]

**Extended Material and Methods**

**Drosophila genetics and mutant generation**

The DrosDel (1) isogenic *w^1118^* (*iso w^1118^*) wild type was used as a common genetic background for all genotypes. *iso w^1118^* was used as wild-type control flies. *ΔAMP14* flies lacking the 14 classical AMP genes from the *Defensin*, *Drosocin*, *Attacin*, *Diptericin*, *Metchnikowin*, *Drosomycin*, and Cecropin gene families were generated as described in Carboni et al. and Hanson et al. (2) (3), and *iso* *Relish^E20^* and *spz^rm7^* flies used as immune deficient controls are the same as used in Hanson et al. (3). *LysB-P^∆^* mutant flies were provided by Shu Kondo and generated through use of two CRISPR gRNAs (gRNA#1 GTGCGATAACTCCCTATCAG, gRNA#2 GTGGCTTTCCCATACAATGT) according to Kondo and Ueda (4) generating a small genomic deficiency on the 3L chromosome. A description of the locus is given in **Fig S1**. *LysB-P^∆^* were then isogenized as described by Ferreira et al. (5).

Mixed male and female embryos were initially collected and reared as larvae, and both males and female adults were maintained together over the course of experiments. At specified sample time points, only female flies were collected and analyzed to avoid complications arising from potential sex-specific effects.

**16S rRNA gene amplicon sequencing and data processing**

Divisive Amplicon Denoising Algorithm 2 (DADA2) pipeline (“dada2” package version 1.14.1 in R) was used to process the sequencing data. All functions were run using the recommended parameters (<https://benjjneb.github.io/dada2/tutorial.html>) except for “expected errors” during the filtering step which was set to (maxEE=2,5) in “filterAndTrim” function. The RDP database was used for taxonomy assignments. Downstream analyses were performed in R version 3.6.0. Reads belonging to mitochondria, chloroplasts, and eukaryotes were excluded from further analyses (“phyloseq” package version 1.30.0, “subset_taxa” function (6). Only the variants present in at least 10 samples with a total of 100 reads were retained for downstream analyses (“genefilter” package version 1.68.0, “filterfun_sample” function (7). To complement the taxonomic classification based on the RDP database, sequence variants were further assigned to the gut microbiota members based on their alignment to the full 16S rRNA gene sequences obtained by Sanger sequencing of each isolate.

**Gnotobiotic fly cultivation and media**

To generate germ-free flies, embryos were collected from an overnight egg laying on grape juice-agar plates supplemented with yeast. Embryos were washed with tap water, sterilized by soaking in 3% bleach for 3 minutes and were rinsed with autoclaved water 3 times. ~200 eggs were counted on a mesh under a laminar flow hood and were transferred to filter cap falcon tubes (TPP catalogue #87050) containing the autoclaved larval medium (0.79% Agar, 5.2% cornmeal, 11% sucrose, 4% yeast, 1.12% Moldex, and 0.77% propionic acid, where the last two were added when the medium was <78˚C) supplemented with antibiotics (50µg/µl Ampicilin, 50µg/µl Kanamycin, 10µg/µl Erythromycin, 10µg/µl Tetracyclin).

Upon emergence, 0-2 day old adults were transferred to autoclaved adult medium (0.79% Agar, 5.2% cornmeal, 11% sucrose, 2% yeast, 1.12% Moldex, and 0.77% propionic acid, where the last two were added when the medium was <78˚C) without antibiotics to remain germ-free or to be colonized with commensals. To colonize adults, cultured bacteria were centrifuged at 3000 rpm for 10 minutes and resuspended in sterile PBS to a concentration of ~5 x 10^6^ cells per 100µl. To generate the commensal cocktail, 100µl of each bacterial suspension was mixed in 1:1 ratio to maintain the same cell concentration. 50µl of the bacterial suspension was spread over the adult medium using glass beads (3mm diameter) for 10 seconds and the tubes were air dried under the laminar hood for 2 hours. Germ-free adults raised in antibiotic medium were anaesthetized on ice for 10 minutes, and 20-30 adults were transferred to each tube.

**Peptidoglycan digestion assay**

Gut extracts from wild-type flies (*w*) and lysozyme mutants (*LysB-P^∆^)* were mechanically lysed by bead beating in 1x PBS. 10 µl of 100µg/ml protein fly lysate was incubated for 48h at 37°C with 500 rpm agitation with 10 µl PGN of 5 mg/ml PGN purified from *E.faecalis* as described in Leulier et al., 2003 (8). As a positive control 10ul of hen egg lysozyme (Sigma) at 100µg/ml final concentration was incubated with 10 µl of PGN from *E.faecalis* for the same amount of time. Lysozyme activity was monitored by measuring the optical density at OD 450 after 24h and 48h of incubation.

**Extended Bibliography**

1. Ryder E, Blows F, Ashburner M, Bautista-Llacer R, Coulson D, Drummond J, Webster J, Gubb D, Gunton N, Johnson G, O’Kane CJ, Huen D, Sharma P, Asztalos Z, Baisch H, Schulze J, Kube M, Kittlaus K, Reuter G, Maroy P, Szidonya J, Rasmuson-Lestander Å, Ekström K, Dickson B, Hugentobler C, Stocker H, Hafen E, Lepesant JA, Pflugfelder G, Heisenberg M, Mechler B, Serras F, Corominas M, Schneuwly S, Preat T, Roote J, Russell S. 2004. The DrosDel collection: A set of P-element insertions for generating custom chromosomal aberrations in Drosophila melanogaster. Genetics 167:797–813.

2. Carboni A, Hanson MA, Lindsay SA, Wasserman SA, Lemaître B. 2021. Cecropins contribute to Drosophila host defence against fungal and Gram-negative bacterial infection. bioRxiv 2021.05.06.

3. Hanson MA, Dostálová A, Ceroni C, Poidevin M, Kondo S, Lemaître B. 2019. Synergy and remarkable specificity of antimicrobial peptides in vivo using a systematic knockout approach. Elife 8:e44341.

4. Kondo S, Ueda R. 2013. Highly improved gene targeting by germline-specific Cas9 expression in Drosophila. Genetics 195:715–721.

5. Ferreira ÁG, Naylor H, Esteves SS, Pais IS, Martins NE, Teixeira L. 2014. The Toll-Dorsal Pathway Is Required for Resistance to Viral Oral Infection in Drosophila. PLoS Pathog 10 (12) :e1004507.

6. McMurdie PJ, Holmes S. 2013. Phyloseq: An R Package for Reproducible Interactive Analysis and Graphics of Microbiome Census Data. PLoS One 8:e61217-e61217.

7. Gentleman R, Carey V, Huber W, Hahne F. 2018. Genefilter: methods for filtering genes from high-throughput experiments. R package version 1.62. 0. Available here https//bioconductor org/packages/release/bioc/html/genefilter html Accessed June.

8. Leulier F, Parquet C, Pili-Floury S, Ryu JH, Caroff M, Lee WJ, Mengin-Lecreulx D, Lemaitre B. 2003. The Drosophila immune system detects bacteria through specific peptidoglycan recognition. Nat Immunol 4:478–484.
